# Improving adherence to surveillance and screening recommendations for people with colorectal cancer and their first degree relatives: a randomized controlled trial

**DOI:** 10.1186/1471-2407-12-62

**Published:** 2012-02-08

**Authors:** Mariko Carey, Rob Sanson-Fisher, Finlay Macrae, David Hill, Catherine D'Este, Christine Paul, Christopher Doran

**Affiliations:** 1 The Priority Research Centre for Health Behaviour, School of Medicine and Public, Health Faculty of Health, The University of Newcastle, Callaghan, New South Wales 2308, Australia; 2 Dept of Colorectal Medicine & Genetics, Level 3 Centre, City Campus, The Royal Melbourne Hospital, Grattan Street, Parkville, Victoria 3050, Australia; 3 The Cancer Council Victoria, 1 Rathdowne Street, Carlton, Victoria 3053, Australia

**Keywords:** Cancer, Colorectal cancer, Early detection, Screening, Surveillance, Guideline adherence

## Abstract

**Background:**

Colorectal cancer (CRC) is among the leading causes of cancer-related morbidity and mortality worldwide. Despite clinical practice guidelines to guide surveillance care for those who have completed treatment for this disease as well as screening for first degree relatives of people with CRC, the level of uptake of these recommendations remains uncertain. If outcomes for both patients and their families are to be improved, it is important to establish systematic and cost-effective interventions to improve adherence to guideline recommendations for CRC surveillance and screening.

**Methods/Design:**

A randomized controlled trial will be used to test the effectiveness of a print-based intervention to improve adherence to colonoscopy surveillance among people with CRC and adherence to CRC screening recommendations among their first degree relatives (FDRs). People diagnosed with CRC in the past 10 months will be recruited through a population-based cancer registry. Consenting participants will be asked if their first degree relatives might also be willing to participate in the trial. Information on family history of CRC will be obtained from patients at baseline. Patients and their families will be randomized to either minimal ethical care or the print-based intervention. The print-based intervention for FDRs will be tailored to the participant's level of risk of CRC as determined by the self-reported family history assessment. Follow up data on surveillance and screening participation will be collected from patients and their FDRs respectively at 12, 24 and 36 months' post recruitment. The primary analyses will relate to comparing levels of guideline adherence in usual care group versus print-based group in the patient sample and the FDR sample respectively.

**Discussion:**

Results of this study will provide contribute to the evidence base about effective strategies to a) improve adherence to surveillance recommendation for people with CRC; and b) improve adherence to screening recommendation for FDRs of people with CRC. The use of a population-based cancer registry to access the target population may have significant advantages in increasing the reach of the intervention.

**Trial registration:**

This trial is registered with the Australian and New Zealand Clinical Trials Registry Registration Number (ACTRN): ACTRN12609000628246.

## Background

Colorectal cancer (CRC) is among the most prevalent cancers worldwide and carries a substantial mortality and morbidity burden [1]. Five year survival ranges between 8% and 93%, depending on stage of disease [2]. Among people who have undergone surgical resection for CRC, surveillance colonoscopy is recommended at regular intervals to detect recurrence and metachronous cancers. In Australia, the National Health and Medical Research Council (NHMRC) clinical practice guidelines recommend that colonoscopy is performed at the time of diagnosis, or if this is not possible, within six months' of surgery, and then at an interval of 3-5 years [3]. Although NHMRC guidelines [3] have been available for some time, the level of uptake of the recommendations is uncertain. Australian studies have indicated that between 23%-38% of surveillance colonoscopies are in line with NHMRC recommendations [4,5].

Screening of first degree relatives (FDRs) of patients with colorectal cancer is widely recommended [6]. Evidence for the benefits of screening of first degree relatives of colorectal cancer patients come from prospective and retrospective cohort studies [5,7-9] and from a non-randomized study of relatives in a high risk familial setting, where there was a reduction in mortality from colorectal cancer in those accepting compared to those refusing screening [1]. The type of screening and recommended interval is dependent upon level of risk [3]. Current Australian guidelines classify risk levels for FDRs as shown in Table 1[3]. Recommended screening for each of these risk categories is as follows: *Risk level 1*: Faecal occult blood testing every second year from age 50 years and consider flexible sigmoidoscopy every 5 years. *Risk level 2: *Colonoscopy every 5 years starting at age 50 years or at 10 years younger than the earliest age of first diagnosis of bowel cancer in the family, whichever comes first. Flexible sigmoidoscopy and double contrast barium enema or CT colonography may be offered where colonoscopy is contraindicated. *Risk level 3*: Colonoscopy every one or two years for families with proven Hereditary Non-polyposis Colorectal Cancer (HNPCC) commencing at 25 years or 5 years before the earliest age of cancer diagnosis in the family, whichever comes first; annual screening for proven gene carriers or clinically affected members of Amsterdam-positive families.

**Table 1:** Risk categories for colorectal cancer based on family history

Risk Category	Characteristics
Category 1. At or slightly above average risk	Asymptomatic people who have: • no personal history of bowel cancer, advanced adenoma, or chronic ulcerative colitis, and • either no close relatives with bowel cancer or one first-degree or second-degree relative with bowel cancer diagnosed at age 55 years or older.
Category 2. Moderately increased risk	Asymptomatic people who have: • one first-degree relative with bowel cancer diagnosed before the age of 55 years (without the potentially high-risk features listed below for category 3), or • two first-degree *or *one first- and one second-degree relative(s) on the same side of the family with bowel cancer diagnosed at any age (without the potentially high-risk features listed below for category 3).
Category 3. Potentially high risk	Asymptomatic people who have: • three or more first-degree or a combination of first-degree and second-degree relatives on the same side of the family diagnosed with bowel cancer (suspected HNPCC), or • two or more first-degree or second-degree relatives on the same side of the family diagnosed with bowel cancer, including any of the following high-risk features: - multiple bowel cancers in the one person - bowel cancer before the age of 50 years - at least one relative with cancer of the endometrium, ovary, stomach, small bowel, renal pelvis, ureter, biliary tract or brain, or at least one first-degree relative with a large number of adenomas throughout the large bowel (suspected FAP), or • somebody in the family in whom the presence of a high-risk mutation in the APC (adenomatous polyposis coli) gene or one of the mismatch repair (MMR) genes has been identified.

• The above definitions are based on the National Health and Medical Research Council's clinical practice guidelines [3].

While a small number of studies have attempted to improve adherence to screening among FDRs [10], few studies have evaluated the effectiveness of systematic interventions which target care for both the person with cancer and their FDRs. An improvement in guideline adherence was demonstrated at a single site following dissemination of the guidelines to all specialists and implementation of a nurse co-ordinator role to ensure that screening/surveillance recommendations matched guideline recommendations [4]. However, effective interventions need to be developed that have the potential for broad implementation and that enable follow up and screening advice to be systematically and comprehensively provided to all identified colorectal cancer patients and their relatives, not just to those who present for care. Therefore, the current study will seek to evaluate an intervention which aims to improve screening/surveillance care for both people with bowel cancer and their first degree relatives using a population-based cancer registry as the reliable access point for all such patients. Thus, the study tests an intervention capable of having an impact on the entire affected population [11].

## Aims

The aims of this trial will be to:

i. Test the effectiveness of tailored print-based intervention to improve a) adherence to surveillance colonoscopy recommendations for people with bowel cancer b) adherence to bowel cancer screening guidelines among their first degree relatives.

ii. Examine how the identified cancer patients' and first degree relatives' socio-demographic characteristics, disease characteristics (for index case), risk category (for first degree relatives), intervention groups and providers' characteristics influence whether or not: a) colorectal cancer patients; and b) first degree relatives of colorectal cancer patients, are appropriately screened.

iii. Undertake a cost-effectiveness analysis of the intervention strategy by comparing the differential cost of an intervention and usual care conditions by changes in the quality-adjusted life years associated with each intervention.

## Methods/Design

### Ethics approvals

Full ethical approval for this study has been obtained from the Cancer Council Victoria Human Research Ethics Committee and the University of Newcastle Human Research Ethics Committee.

### Setting

People with a diagnosis of bowel cancer within the last 10 months will be recruited from population-based cancer Victorian Cancer Registry covering the entire Australian state of Victoria (population 5.5 million).

### Patient eligibility and recruitment

People with bowel cancer (index cases) who meet the following criteria will be identified from the cancer registry: 1) aged 18 or older; 2) registered with cancer registry within 10 months of diagnosis. The registry will write to the notifying doctor of each potentially eligible person and ask the doctor to contact the registry within 4 weeks if there is any reason why the patient should not be contacted about the study. Index cases whose doctors perceive them to be unsuitable for the study will be excluded. The registry will contact remaining index cases and ask their permission to pass their contact details onto the research team. Two reminders will be sent to non responders. Those who consent will be invited to participate in the trial with two reminders sent to non responders.

### Assessment of family history

Patients will complete a baseline computer assisted telephone interview (CATI) to assess family history. On the basis of their report of family history each index case and their family will be classified as 1) average risk; 2) increased risk or 3) potentially high risk. These categories, defined according to the definition of the National Health and Medical Research Council (NHMRC), are each associated with different types of screening recommendations. Participants will also complete questions on age, gender, marital status, education and employment, disease characteristics, treatment, screening history, surveillance intentions and quality of life. Quality of life will be assessed using the European Quality of Life Scale (EQ-5D version) which covers 5 dimensions of health: mobility, self-care, usual activities, pain/discomfort and anxiety/depression [12].

### FDR eligibility

First degree relatives of people with bowel cancer will be eligible to participate in the trial if they are a) aged 18 or older; b) English speaking; and c) able to provide informed consent. Those with a prior diagnosis of bowel cancer, Crohn's disease, or inflammatory bowel disease will be ineligible.

### Recruitment of FDRs

Patients will be asked to provide the names and contact details of any living first degree relatives aged 18 or older. They will be asked to indicate which (if any) of their FDRs they are willing for the research team to contact about the study. A choice will be given regarding method of contact of FDRs. Option one will involve the patient being given a letter about the study which they can pass onto their relatives; while option 2 will involve the research team contacting the relative(s) directly by mail. Patients will be asked to seek their relatives' permission prior to selecting the latter option. Non responders for option one receive one reminder letter. The information pack will contain a cover letter, information statement about the study, and ask the person to contact the research team if they were interested in participating. If the index case has provided the relative's phone number, the letter will ask the relative to return a "do not contact" form within the next two weeks if they did not want a researcher to contact them about the study. FDRs that consent to being contacted will be telephoned and asked to participate in a brief screening interview to assess trial eligibility. Consenting eligible FDRs will complete a baseline computer-assisted telephone interview (CATI) which will include questions about age, gender, marital status, education, employment, knowledge about screening and prior screening history and future intentions of screening, use of complementary therapies, and quality of life. Quality of life will be assessed using the European Quality of Life Scale [12].

### Randomization

Consenting patients and their first degree relatives will be randomized as a family unit to either "minimal ethical care" or a tailored print based intervention. Randomization will be conducted centrally using a computer generated procedure. Interviewers will be blind to participants' allocation.

### Intervention

Patients assigned to the intervention group will receive a letter detailing recommendations for follow up care. A fact sheet with information about recommended follow up care will be mailed to patients. The patient's GP and surgeon will also be sent a fact sheet with information about best evidence surveillance care. First degree relatives will receive a tailored letter which provides advice on recommended bowel cancer screening tests and intervals based on their level of family risk. A brochure detailing the three risk levels and their corresponding screening recommendations will be enclosed. Family risk will be determined based on patient self report information and will correspond to the three levels of risk identified in the National Health and Medical Research Guidelines. The GP of the FDR will also receive a tailored letter indicating the likely risk category of the first degree relative. A brochure detailing screening recommendations for each risk category will be enclosed.

### Minimal ethical care

Patients and first degree relatives assigned to the minimal ethical care group will receive a generic booklet on bowel cancer and generic pamphlet on bowel cancer screening respectively. First degree relatives categorized at very high risk will sent the tailored information as per the intervention group as part of our duty of care.

### Follow up assessments

Both first degree relatives and patients will be mailed follow up surveys at 12, 24 and 36 months. First degree relatives will be asked to provide self report information on the type of bowel cancer screening undertaken in the past 12 months (if any), and any days off work due screening. Patients will be asked to provide self report information about participation in surveillance colonoscopy within the past 12 months, quality of life. Patients or first degree relatives who report having had a colonoscopy or bowel cancer screening test in the past 12 months will be asked for permission to contact their doctor to verify the timing and results of the test. A random sample of 10% of self reported screening will be verified in this way to determine accuracy of self reported screening behavior.

### Statistical analysis

Baseline characteristics of index patients and first degree relatives will be reported for intervention and control groups. *Aim 1*. The proportion of index patients and the proportion of first degree relatives who undertake screening/surveillance tests in line with NHMRC recommendations for their risk category in years 1, 2 and 3 following diagnosis in the index case will be compared between the two experimental groups using the chi-square test. *Aim 2*. Sociodemographic characteristics, screening history, risk status, concurrent engagement in alternative programs of surveillance and symptom status, of those who are appropriately and inappropriately screened at each time point will be compared using the chi-square test for categorical variables and the t-test or a non-parametric equivalent for continuous variables. Logistic regression will then be used to examine factors associated with appropriate screening/surveillance while adjusting for potential confounders. Variables will be included in the initial logistic model if they have a p value of 0.2 or less on univariate analyses, with backward stepwise methods used to exclude variables with a p value of 0.10 or more on the likelihood ratio test. Analysis will be undertaken separately for the index cases and for first degree relatives. Analyses will be adjusted for clustering of relatives within index patients using Generalised Estimating Equations.

### Sample size

Approximately 3000 eligible new cases of colorectal cancer are expected to be identified through the Victorian Cancer Registry each year. Based on previous experience, 3% of clinicians will refuse permission for their patient to be approached, therefore we expect permission to contact 2900. Based on prior research, we expect 60% of patients contacted by the VCR agree to be contacted by the research team and 70% of these consent to participate in the study. Recruitment will be undertaken for 12 months, thus 1200 index cases will be recruited in total, or 600 per group. *Aim 1*. Assuming that 20% of participants will be lost at each follow-up time (leaving 480, 380, 300 index cases per group available at 12, 24, 36 month), the study will have 80% power, with a 5% significance level, to detect a difference of 10% between experimental groups in the proportion of index cases appropriately surveillance at 12 month follow-up, 12% difference at 24 and 36 month follow-up. It is estimated that there will be an average of 2 eligible first degree relatives for each index case, and that 70% of these will consent and be available for 12 month follow-up, with a 20% loss to follow-up at each of the remaining assessment times. This will provide 1680, 1350 and 1050 relatives at 12, 24 and 36 months. Allowing for a design effect of 2 will provide an effective sample size of 400, 330 and 260 per group at each time point, which is adequate to detect a 10% difference in the proportion of first degree relatives appropriately screened at 12 months, 12% at 24 months and 13% at 36 months with 5% significance level and 80-85% power. A 10% difference at the first follow-up is considered to be clinically meaningful, given the burden of disease from colorectal cancer and the intervention effect is anticipated to increase over time, as the intervention will continue for the three year follow-up period. *Aim 2*. Assuming at least one third of individuals will be appropriately screened at each follow up time, the study will also have at least 80% power to detect differences in characteristics of those who are and are not followed up/screened appropriately of 10-13% for binary variables and 0.2-0.25 standard deviations for continuous variables for index cases, and 11-14% for binary variables and 0.22 -0.27 standard deviations for continuous variables for FDRs.

### Cost effectiveness analysis

The cost-effectiveness analysis will examine the null hypothesis that the mean cost-effectiveness of the proposed health care intervention is no different to the mean cost-effectiveness of usual care. The perspective adopted for this analysis is societal and will encapsulate the viewpoint of the health sector in which resources associated with the provision of care (i.e., colorectal screening) are combined with cost-savings arising from that care, and the viewpoint of the patient in terms of out of pocket expenditures associated with treatment. Costs will then be compared with changes in patients' quality adjusted life years.

## Time line for the study

Recruitment to the trial began in 2010 and is expected to be completed in early 2012.

## Discussion

In order to improve health outcomes across a range of areas, there is an urgent need to find effective strategies for improving uptake of and adherence to evidence-based guidelines. Results of this study will provide contribute to the evidence base about effective strategies to a) improve adherence to surveillance recommendation for people with CRC; and b) improve adherence to screening recommendation for FDRs of people with CRC. The use of a population-based cancer registry to access the target population may have significant advantages for increasing the reach of the intervention. If the intervention is found to be effective there is scope for it to be replicated in other population-based cancer registries.

## Competing interests

The authors declare that they have no competing interests.

## Authors' contributions

MC took the lead on drafting the manuscript and has contributed to development of the study protocol. RSF, CDE, FM, DH, and CD conceptualized the project and wrote the grant application, contributed to development of the study protocol and writing of the manuscript. CP assisted in development of the protocol, and writing of the manuscript. All authors read and approved the final manuscript

## Pre-publication history

The pre-publication history for this paper can be accessed here:

http://www.biomedcentral.com/1471-2407/12/62/prepub
